# Creatine kinase in ischemic and inflammatory disorders

**DOI:** 10.1186/s40169-016-0114-5

**Published:** 2016-08-15

**Authors:** David Kitzenberg, Sean P. Colgan, Louise E. Glover

**Affiliations:** 1 Mucosal Inflammation Program, University of Colorado, Anschutz Medical Campus, 12700 East 19th Ave. MS B-146, Aurora, CO 80045 USA; 2 Department of Medicine, University of Colorado Anschutz Medical Campus, Aurora, CO 80045 USA

**Keywords:** Hypoxia, Ischemia, Creatine, Creatine kinase, Phosphocreatine, Energetics, Mitochondria, Metabolism

## Abstract

The creatine/phosphocreatine pathway plays a conserved and central role in energy metabolism. Compartmentalization of specific creatine kinase enzymes permits buffering of local high energy phosphates in a thermodynamically favorable manner, enabling both rapid energy storage and energy transfer within the cell. Augmentation of this metabolic pathway by nutritional creatine supplementation has been shown to elicit beneficial effects in a number of diverse pathologies, particularly those that incur tissue ischemia, hypoxia or oxidative stress. In these settings, creatine and phosphocreatine prevent depletion of intracellular ATP and internal acidification, enhance post-ischemic recovery of protein synthesis and promote free radical scavenging and stabilization of cellular membranes. The creatine kinase energy system is itself further regulated by hypoxic signaling, highlighting the existence of endogenous mechanisms in mammals that can enhance creatine metabolism during oxygen deprivation to promote tissue resolution and homeostasis. Here, we review recent insights into the creatine kinase pathway, and provide rationale for dietary creatine supplementation in human ischemic and inflammatory pathologies.

## Introduction

Creatine (Cr) plays a pivotal role in cellular energy homeostasis, particularly in tissues with highly dynamic energy demands such as the brain, striated muscle and the gut. Within the cell, creatine and its associated enzyme creatine kinase (CK) facilitate the shuttling of high energy phosphates in the form of phosphocreatine (PCr) between sites of ATP generation, i.e. mitochondrial oxidative phosphorylation and glycolysis, and compartmentalized ATP consumption. As such, the Cr/CK system defines an important and highly conserved phosphagen circuit, providing support for mitochondrial respiration and cellular energy turnover by mitigating temporal and spatial imbalances in ATP supply and demand [1].

Vertebrates express four distinct CK isozymes, and patterns of expression vary by tissue and by developmental stage. Cytosolic muscle creatine kinase (CKM) is expressed primarily in sarcomeric skeletal and cardiac muscle cells, while the ubiquitous cytosolic brain isoform (CKB) is found in most non-muscle tissues. Two mitochondrial CK (mtCK) isoforms have also been characterized, namely muscle-specific sarcomeric (smtCK) and ubiquitous mtCK, both of which localize to the mitochondrial intermembrane space [2]. Temporal and tissue-specific CK expression is transcriptionally regulated by a number of factors, including myocyte-specific enhancer binding factor 2 (MEF-2) [3, 4], myogenic differentiation factor D (MyoD) [5], specificity protein 1 (Sp1) [6] and hypoxia-inducible factor (HIF) [7]. Moreover, substantial evidence from in vitro studies suggests that cytosolic and mitochondrial CK expression is modulated by estrogen receptor-mediated gene activation [8–10].

All CK isozymes catalyze the reversible transfer of γ-phosphate from ATP to the guanidino group of Cr to generate PCr and ADP, thus mediating efficient cytosolic storage of high-energy phosphates for rapid, focal ATP replenishment. The Cr/CK circuit is tightly linked to mitochondrial structure and energetics, as MitCK is coupled to ATP export via the adenine nucleotide transporter (ANT), and to ATP synthesis and respiratory chain activity. This mitochondrial coupling reduces reactive oxygen species (ROS) generation and inhibits mitochondrial permeability transition, an early event in apoptosis. The Cr/CK reaction also modulates intracellular pH to protect cells from damage associated with internal acidification and ATP depletion [11, 12]. Furthermore, PCr can interact with and protect cellular membranes [13], while Cr has been shown to scavenge free radicals and to harbor antioxidant properties [14, 15].

Dietary Cr supplementation increases the intracellular pool of Cr and PCr available for ATP generation, thereby supporting overall cellular energy homeostasis. As a nutritional supplement, Cr retains an excellent safety profile even in aged individuals with chronic disorders, and has been widely used by athletes in recent decades as an ergogenic aid to improve muscle performance. Additional evidence from recent studies supports the use of Cr supplementation as an adjuvant therapy in a diverse spectrum of disorders, whose central pathologies converge on a dysregulation of cellular bioenergetics. This review outlines our current understanding of the Cr/CK system in cellular energy homeostasis, and discusses recent compelling evidence for the beneficial effects of Cr supplementation in inflammatory and ischemic disorders.

## Disorders of creatine metabolism and transport

The daily Cr requirement for an average 70-kg adult male is approximately 2 g, up to half of which may be derived by intestinal absorption from dietary sources such as meat, fish and other animal products [16]. The remainder is synthesized de novo from arginine, glycine and methionine by a two-step enzymatic reaction, utilizing arginine:glycine aminotransferase (AGAT) to generate guanidinoacetic acid (GAA) predominantly in the kidney, followed by hepatic methylation of GAA via guanidinoacetate methyltransferase (GAMT), using *S*-adenosylmethionine (SAM) as a methyl donor [17]. Cr biosynthesis accounts for ~70 % of the total utilization of labile methyl groups in the body, with SAM availability governed by levels of folate, vitamin B12, vitamin B2 and one-carbon precursors such as serine, histidine, tryptophan and choline [18, 19]. As such, deficiencies in folic acid and/or vitamin B12 are proposed to undermine GAA methylation and impact Cr biosynthesis [20]. Dietary and endogenous Cr enters systemic circulation and is actively transported into multiple tissue compartments by a transmembrane Na^+^ and Cl^−^ -dependent creatine transporter (CrT), encoded by the *SLC6A8* gene [21]. Approximately 1.5–2.0 % of the total Cr and PCr pool is degraded daily via simple non-enzymatic chemical dehydration to creatinine, which is lost from cells by diffusion and targeted for urinary excretion [22].

Heritable defects in Cr biosynthesis (*AGAT, GAMT*) and Cr transport (*SLC6A8*) have been identified and are broadly characterized as Cr deficiency syndromes. Mutations in *AGAT* and *GAMT* display an autosomal recessive pattern of inheritance, and deficiency in either of these enzymes manifests in developmental delay or regression, mental retardation, seizures and severe disturbance of expressive and cognitive speech [23]. Importantly, early detection and intervention with high dose Cr supplementation favorably impacts neurodevelopmental outcomes in affected individuals [24]. Polymorphisms in *SLC6A8* gene encoding the Cr transporter are a primary cause of X-linked mental retardation with a prevalence of 2 % in affected males, and have also been described in males with idiopathic mental retardation [25, 26]. Additional features of Cr transporter deficiency include delayed speech and language development with mild to moderate motor dysfunction, including extrapyramidal movement abnormalities. Interestingly, gastrointestinal problems such as neonatal feeding difficulties, vomiting and failure to thrive are also frequently associated, and among the earliest symptoms described [27]. These clinical observations suggest that Cr is imperative not only to cerebral function in the central nervous system, but also to gastrointestinal function and homeostasis.

## Creatine, the intestinal barrier and inflammatory bowel disease

Cytosolic CKB is prominently expressed in smooth muscle and epithelial cells of the human intestine [28], and immunolocalization studies indicate retention of the Na^+^Cl^−^ dependent Cr transporter selectively to the enterocyte apical membrane [29]. The mode of intestinal Cr absorption in humans remains somewhat unclear, as transepithelial transport of Cr would necessitate a basolateral membrane transporter that is not coupled to Na^+^Cl^−^. A second Cr transporter, the monocarboxylate transporter 12 (MCT12), has recently been identified as a potential candidate for intestinal epithelial basolateral Cr transport [30]. Alternatively, intestinal Cr absorption may occur via paracellular movement by solvent drag transport, such that apical Cr uptake by epithelial cells is directed exclusively towards epithelial bioenergetics [31]. Recent findings have highlighted an important role for the Cr/CK shuttle in intestinal hypoxia and inflammation [7]. While this work focused largely on intestinal epithelial cells, additional studies indicate that gut homeostasis may also be modulated by Cr/CK bioenergetics in the intestinal immune cell repertoire and Cr metabolism by gut microbiota.

### Intestinal epithelial cells

**Fig. 1:**
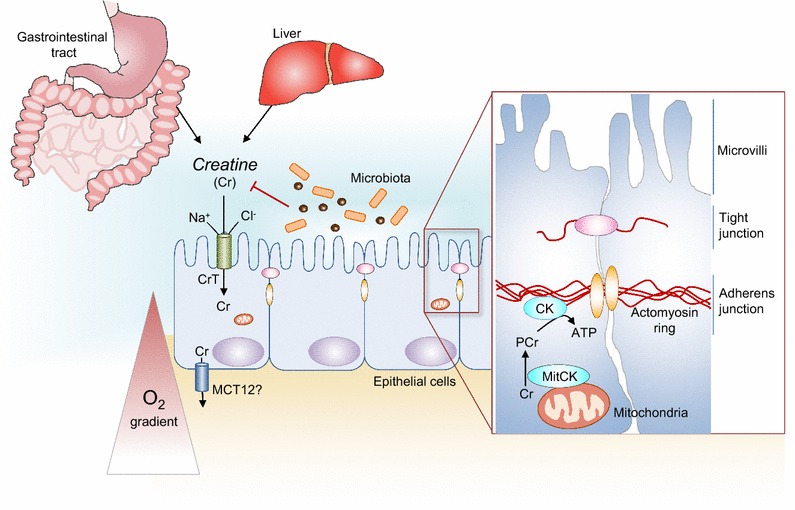
Cr/CK shuttle and the intestinal mucosal barrier. Cr is derived from dietary sources in the gastrointestinal tract, or by de novo synthesis synthesis primarily in the liver. The Na^+^ and Cl^−^-dependent creatine transporter (CrT) is expressed in the apical membrane of intestinal epithelial cells, facilitating Cr uptake from the gut lumen. Although intestinal Cr absorption in humans has not been well characterized, potential routes for Cr absorption into systemic circulation include paracellular movement by solvent drag transport, or via basolateral Cr transport by the monocarboxylate transporter 12 (MCT12). Gut microbiota express specific enzymes that can mediate Cr and creatinine breakdown. In hypoxic intestinal epithelial cells, cytosolic CK localizes to apical adherens junctions in complex with the actomyosin cytoskeletal network, providing a conduit for rapid ATP generation during the energy-dependent processes of epithelial junction assembly and barrier restitution

Intestinal epithelia that line the gut mucosa constitute the primary cellular barrier against the external luminal environment. This highly dynamic barrier is intricately regulated by myriad factors, including local oxygen tension, to both accommodate nutrient and fluid transport and exclude antigenic material [32]. Intestinal epithelia are polarized, with apical surface features such as mucus secretion and intercellular junctions that are optimized for luminal interaction and enteric microbe exclusion. Within epithelia and other polarized cells, where mitochondria are located at a distance from subcellular regions of ATP consumption, differentially localized CK isozymes have been shown to facilitate a high-energy PCr/Cr circuit [11]. Early studies of CK in intestinal epithelia showed that while mitochondria and resident mtCK were excluded from brush borders by a dense cytoskeletal network, CKB localized to the brush border terminal web [33, 34]. Moreover, functional coupling between CKB and myosin II at the circumferential actomyosin ring was found to confer a spatial selective energetic advantage for myosin ATPase activity, mediating the static tension and contractility of actin filaments. The cytoskeletal network that supports apical epithelial junctions is among the most highly ordered arrays of actin filaments in nature [35]. This actomyosin network mediates selective barrier function in health and disease [36] and is a primary target for molecular remodeling by diverse inflammatory stimuli [37]. Recent work showed that myosin II and cytosolic CKs are highly enriched at the apical adherens junction of polarized intestinal epithelial cells, and pharmacological inhibition of CK markedly disrupts apical junction assembly and barrier integrity. Cytoskeletal and apical junction rearrangements that permit epithelial turnover and transepithelial transport are energy-dependent processes, and as such, structurally associated CK is poised to function as a conduit for rapid ATP generation in mucosal barrier dynamics (Fig. 1) [7].

Barrier dysregulation is a prominent determinant of susceptibility to inflammatory bowel disease (IBD). IBD encompasses a spectrum of chronic intestinal inflammatory disorders with increasing global prevalence, with Crohn’s disease and ulcerative colitis comprising the predominant entities [38]. The mucosal barrier is subject to an austere oxygenation profile even under steady-state conditions, and intestinal inflammation incurs prodigious metabolic shifts and further depletion of local oxygen, culminating in hypoxic lesions. As such, hypoxia predominates normal intestinal metabolism and barrier regulation during both homeostasis and active inflammation. Cellular programming by hypoxia-inducible factor (HIF) has been shown to tonally regulate epithelial homeostasis, and to promote barrier function under inflammatory conditions associated with IBD [32, 39, 40]. We recently examined the differential contribution of HIF-1α and HIF-2α to transcriptional changes in intestinal epithelia. For these purposes, we performed chromatin immunoprecipitation (ChIP) with HIF-1α and HIF-2α antibodies followed by hybridization to a promoter microarray. Highly enriched subsets of HIF-1α ChIP hits included multiple enzymes of the glycolytic pathway, autophagic targets and jumonji domain (JmjC) containing histone demethylases. In addition, this analysis revealed prominent changes associated with metabolism, immunity and transcription. It is notable that promoter sequences for *CKB* and *CKM* genes emerged as high fidelity HIF-2 selective targets. Likewise, both mitochondrial isoforms of CK as well as the major creatine transporter SLC6A8 were significantly enriched in HIF-2 ChIP. These studies also demonstrated that dietary supplementation with 2 % Cr provided marked protection in two mouse models of experimental IBD.

Importantly, intestinal expression of mitochondrial and cytosolic CK enzymes was shown to be attenuated in clinical IBD biopsies. Reduced ATP levels have also been observed in inflamed tissue from patients with IBD [41], and non-inflamed biopsies obtained from Crohn’s disease patients are more sensitive to uncoupling of oxidative phosphorylation [42]. These observations are particularly noteworthy, as chronic inflammation and the altered tissue metabolic profile associated with IBD is an established major risk factor for colitis-associated colorectal cancer. The attenuated expression of CK enzymes in IBD tissue suggests that intestinal Cr metabolism and PCr/CK energetics may be compromised in at least a subset of IBD patients, and several studies have identified reduced levels of CKB in colonic tumors [43, 44]. Moreover, overexpression of dominant negative CKB mutants was found to promote an epithelial-to-mesenchymal transition (EMT) in colon cancer cells [45]. Overall, these findings indicate that impaired Cr/PCr shuttling may contribute to dysregulated mitochondrial energetics and the increased barrier permeability characteristic of inflamed mucosae. Most notably, this work highlights the potential for Cr supplementation in IBD to promote epithelial restitution and ameliorate mucosal inflammation.

### Immune cells

Given the observation of dysregulated CK bioenergetics in IBD and the intimate integration of intestinal epithelia with mucosal immune cells, an interesting correlate is the influence of Cr and CK on immune cell metabolism and effector function in IBD and other inflammatory diseases. The Cr/CK circuit in intestinal immune cell homeostasis remains largely uncharacterized, but several studies support a central role for Cr signaling in phagocytic function and T cell development. PCr and CK isoforms have been identified in mouse resident tissue and inflammation-elicited peritoneal macrophages, as well as in human monocyte-derived macrophage cultures [46]. CKB activity in macrophages has been shown to regulate complement-induced F-actin assembly events in early phagocytosis, likely by providing focal ATP for cytoskeletal rearrangements [47, 48]. CKB has been implicated in metabolic regulation of T lineage cells, promoting activation, proliferation and cytokine secretion [49]. CK is also a component of platelets [46], and CKB binding to the thrombin receptor PAR-1 is thought to provide high energy phosphates for efficient receptor signal transduction during cytoskeletal reorganization [50]. Moreover, CK has recently been shown to dose-dependently inhibit ADP-induced platelet aggregation [51]. The relevance of this is underscored by clinical findings that IBD patients have an increased risk of thromboembolic complications and display abnormalities in thrombin generation, platelet activation and function [52, 53].

### Gut microbiota

The mammalian gastrointestinal tract is host to a diverse microbial ecosystem that helps to shape host immunity and metabolism, as well as support epithelial barrier function. Several lines of evidence suggest that degradation of Cr and creatinine by gut microbiota may impact host physiology and pathology. In contrast to the non-enzymatic conversion of PCr and Cr to creatinine that occurs in vertebrates, a growing number of microorganisms have been shown to express specific enzymes such as creatinine deaminase and creatine amidinohydrolase that mediate creatinine and Cr breakdown. In several *Bacillus*, *Clostridia* and *Escherichia* strains, creatinine is degraded solely to 1-methylhydantoin and ammonia via creatinine deaminase, while in some *Pseudomonas, Brevibacterium* and anaerobic *Clostridia* species, 1-methylhydantoin can be degraded further to sarcosine and glycine [19]. Studies in uremic patients with highly elevated serum creatinine indicate that diffusion of creatinine into the gut lumen can induce microbial creatinine amidohydrolase, creatinine deaminase and Cr amidinohydrolase activity, resulting in creatinine metabolism and partial recycling of Cr [54, 55]. Interestingly, gut microbial taxonomic profiling in a mouse model of senescence recently revealed luminal Cr degradation as a prominent, overrepresented bacterial-encoded signature in older frail mice [56]. This finding is particularly intriguing in light of clinical studies that show Cr supplementation in the elderly promotes muscle strength and hypertrophy [57], and protects against age-related progressive muscle wasting (sarcopenia) [58]. Whether the dysbiosis that is characteristic of IBD can alter Cr metabolism in the gut has yet to be elucidated, but may define an important and previously overlooked axis of host-microbial crosstalk in intestinal inflammation.

## Creatine and perinatal hypoxic ischemic injury

Oxygen deficit or asphyxia at birth is a significant perinatal complication that can result in neurodegeneration, cognitive and behavioral disturbances or neonatal mortality [59]. Secondary to birth asphyxia, postnatal manifestation of hypoxic-ischemic encephalopathy (HIE) is associated with mild to severe multi-organ damage and development of chronic pathologies. Acute maternal intrauterine infection, premature delivery and multiple births are the most frequent natural risk factors leading to fetal or neonatal hypoxia [60]. Primary mechanisms of neurological damage include mitochondrial dysfunction, excitotoxic injury, impaired energy metabolism and oxidative stress. Experimental studies in animal models of birth hypoxia have identified a striking, markedly protective effect of maternal Cr supplementation during pregnancy on neonatal outcomes [61], and argue for the use of dietary Cr in pregnancy as a potential prophylactic therapy or adjunct to conventional treatments in at-risk obstetric populations.

Cr is thought to be actively transported across the placenta from maternal circulation during pregnancy, with placental Cr transporter expression evident as early as 13 weeks of gestation [62]. Expression of mitochondrial and cytosolic CK enzymes in the placenta is highly coordinated and markedly increased in the third trimester, concomitant with increased metabolic activity of the placenta in late pregnancy [63]. It remains unclear when the human fetal reno-hepatic axis for endogenous Cr synthesis is established, although studies in the precocial spiny mouse indicate that this axis develops late in gestation [64]. Thus, the current paradigm implies an absolute requirement for fetal Cr transfer from placental Cr pools likely until late in fetal development, and suggests that infants born pre-term may have diminished capacity for Cr synthesis [61].

Initial studies of Cr-elicited protection in neonatal hypoxic/reperfusion injury focused on rodent fetal brain slices [65, 66] and neonatal rats [67]. These studies described sustained ATP turnover, reduced neuronal cell injury and enhanced post-ischemic recovery of protein synthesis with Cr pre-treatment. Cr has been shown to more readily access the fetal rodent brain than the mature adult brain, possibly due to non-carrier mediated diffusion [68] and/or higher levels of creatine transporter in endothelial cells of the choroid plexus [69]. Studies in the spiny mouse strain demonstrated that maternal dietary supplementation with 5 % Cr (approximately 1.36 g/kg/day) from mid-pregnancy onwards promoted neonate survival and postnatal growth after intrapartum hypoxic insult [70]. In this model, the neuroprotective capacity of Cr in the hypoxic perinatal brain is attributed to reduced lipid peroxidation and apoptosis, likely through maintained mitochondrial function [71]. Importantly, supplementation during pregnancy also enhanced Cr levels in embryonic peripheral organs known to be particularly susceptible to the global oxygen deprivation associated with perinatal asphyxia, supporting the systemic protective effect of Cr in fetal tissues. For instance, Cr loading in utero was found to markedly attenuate hypoxia-induced contractile dysfunction and fiber atrophy of the diaphragm muscle in the spiny mouse [72, 73]. Similarly, birth asphyxia led to disruption of neonatal renal architecture and increased levels of the early kidney injury marker NgaI, all of which were prevented by maternal dietary Cr supplementation [74]. In light of the recently described role for Cr and hypoxic signaling in the gut, a pertinent question is whether maternal Cr supplementation may also protect against necrotizing enterocolitis (NEC), a severe intestinal disorder prevalent in low birth weight, preterm infants [75]. Although the etiology of NEC is incompletely understood, contributing factors are thought to include immature intestinal motility and barrier function, inappropriate initial gut microbial colonization and perinatal hypoxia/ischemia [76]. As such, NEC may define a novel candidate in a group of neonatal pathologies for which maternal Cr supplementation may prove beneficial.

## Creatine in ischemic stroke

In addition to the fetal neuroprotective effects described above, experimental studies have demonstrated that prophylactic Cr treatment is also widely neuroprotective in adult brain tissue against acute anoxic and ischemic cell damage that occurs in ischemic stroke and other cerebrovascular disorders. Early clinical studies in acute stroke patients revealed depletion of Cr and PCr in infarcted cerebral regions, suggesting that abrogated Cr/CK bioenergetics may contribute to the pathogenic features of ischemic cerebral injury such as acidosis, ROS generation and cell death [77]. Studies employing adult rat hippocampal slices demonstrated that Cr pre-incubation mediates a dose-dependent increase in intracellular PCr, as well as delayed anoxic depolarization and protection against anoxia-induced impairment of protein synthesis and neuronal cell death [78, 79]. Dietary Cr pre-treatment in an experimental mouse stroke model was reported to mitigate ischemic neuronal cell death in part by inhibiting cytochrome *c* release and subsequent caspse-3 activation, possibly via primary buffering of ATP levels [80]. Mice pre-treated with oral Cr also showed faster recovery of cerebral blood flow during reperfusion after transient focal cerebral ischemia, likely as a result of enhanced dilator responses to extra-luminal potassium and acidosis [81].

A recent clinical study has now extended these observations to human subjects [82]. Oral Cr supplementation (20 g/day for 7 days) in healthy young adults was found to augment neural Cr stores, increase corticomotor excitability and prevent cognitive decline during acute oxygen deprivation. This study provides compelling in vivo evidence that Cr may act as a neuroprotective supplement under conditions of compromised cellular oxygenation and bioenergetics. As outlined above, HIF is the primary ubiquitous mechanism for adaptive transcriptional responses to oxygen deprivation. Indeed, pharmacological HIF stabilization has been shown to reduce brain tissue injury and edema formation in ischemic stroke [83, 84], while transgenic deletion of HIF-1α augments brain injury in models of neonatal hypoxia–ischemia [85, 86]. An important question, therefore, is how Cr supplementation may integrate with HIF-mediated transcription and enhanced neuronal CK expression in the setting of cerebral hypoxia or ischemia.

## Creatine, CK and ischemic cardiovascular disease

The Cr/CK reaction is the major energy reserve of cardiac muscle cells, and myofibrillar ATP delivery is absolutely required to fuel normal contractile function. As such, a prevailing theory of heart failure posits that the failing heart is “energy-starved”. Indeed, impairment of the CK shuttle during heart failure has been recognized since 1939 [39, 87] and reductions in Cr and CK activity have since been identified in most forms of clinical and experimental heart failure, regardless of pathogenesis [88, 89]. Cr loss in the failing heart is attributed in part to down-regulation of the cardiac creatine transporter [90], as a consequence of post-translational modification [91]. The energetic state of the heart is commonly reported as the PCr/ATP ratio, with a value of ~1.8 in the normal heart. Notably, ATP is maintained near normal levels until end-stage heart failure, due to the buffering capacity of PCr and the CK equilibrium constant strongly favoring ATP synthesis. Multiple studies have shown a reduced PCr/ATP ratio in patients with dilated cardiomyopathy [92, 93], and prior to overt cardiac dysfunction in hypertension [94], obesity [95] and type 2 diabetes [96], suggesting a close association between cardiac energetic status and function.

Myocardial ischemia is characterized by restriction of blood and oxygen supply to the myocardium. In its classic manifestation, cardiac ischemia results from occlusion or narrowing of a coronary artery, inducing tissue hypoxia and rapid depletion of PCr and ATP levels [97]. Stress-induced reduction of the PCr/ATP ratio in female patients with chest pain consistent with myocardial ischemia was found to be a strong predictor of future cardiovascular events [98]. In vivo studies in patients with prior myocardial infarction using phosphorous magnetic resonance spectroscopy ([31] P-MRS) showed that CK flux is reduced in ischemic myocardium, commensurate with the extent of infarct transmurality [99].

Evidence exists to indicate that augmentation of CK and its reactants may prove beneficial in ischemic cardiac disease [100]. Hearts from Cr-deficient GAMT knockout mice displayed reduced inotropic reserve and impaired functional recovery following an ischemic episode [101]. Interestingly, PCr treatment reduced necrotic tissue injury and improved contractile function in animal models of coronary artery ligation [102] and ischemia–reperfusion injury [103]. More recently, PCr administration was found to prevent ventricular dysfunction in a rodent model of transient coronary occlusion [104]. In the clinical setting, significant myocardial protection was reported in patients undergoing coronary artery bypass surgery upon administration of exogenous PCr before, during and post-surgery [105]. Moreover, patients treated with intravenous PCr had reduced incidence of both ventricular fibrillation and ventricular tachycardia post myocardial infarction [106]. PCr is not a known substrate of the creatine transporter [107], and there is limited information on actual uptake of PCr by the human heart. However, PCr uptake has been demonstrated in ex vivo perfused rodent hearts [108] and isolated rat mitochondria and liposomes [109]. Furthermore, PCr is proposed to protect the cardiomyocyte sarcolemma [13, 105] and to inhibit platelet aggregation [51, 110], thus likely exerting a beneficial effect in coronary thrombosis.

Proof-of-principle experimental studies utilizing knockin mice have also demonstrated that transgenic overexpression of the cardiac creatine transporter or the muscle CK isoform CKM can elicit marked protection against stress-induced heart injury. Creatine transporter overexpression was reported to protect against ischemia–reperfusion injury, with improved cardiac energetics and delayed mitochondrial permeability transition pore opening in response to oxidative stress. Importantly, the extent of myocardial damage was found to negatively correlate with tissue Cr levels [111]. Transgenic CKM overexpressing mice were found to maintain CK flux at higher levels in a heart failure model of pressure overload, associated with higher ejection fraction and improved survival [112].

Interestingly, several studies have implicated the HIF pathway in ischemic preconditioning-mediated cardiac protection from ischemic–reperfusion injury [113–115] and prolonged HIF stabilization improves cardiac function in myocardial ischemia [116]. Given the functional regulation of CK metabolism by HIF in the gut, an open question is whether HIF-mediated protection from ischemic damage by ischemic preconditioning may also extend to enhanced CK expression and Cr metabolism in the heart. In sum, while future studies are needed to determine the factors that impair CK energy metabolism and creatine transporter activity in failing or ischemic hearts, current work supports the Cr/PCr pathway as a promising therapeutic target for preventing and treating ischemic cardiovascular disease.

## Conclusions

Although the use of creatine in patients during pregnancy and IBD has yet to be fully evaluated, its profile as a safe nutritional supplement in diverse patient populations is well documented. Creatine has been shown not only to increase muscle mass and prevent age- and disease-related muscle atrophy, but also to enhance overall tissue bioenergetics in a range of pathologies. Clinical evidence strongly supports the profoundly neuroprotective properties of creatine and the beneficial effects of phosphocreatine in cardiovascular stress. Although further work is needed to establish causality, both pre-clinical and clinical studies provide correlative evidence that energetic changes and dysregulation of the Cr/CK pathway are closely linked with the etiology of hypoxic and inflammatory disorders. Altered Cr metabolism by the gut microbiota may define an important influence on the human host creatine pathway, particularly in the context of dysbiosis associated with aging, obesity and IBD. Overall, dietary creatine is a promising candidate as an independent prophylactic treatment or as an adjunct to conventional therapies for ischemic disease.

## Abbreviations

ADP: adenosine diphosphate
AGAT: arginine:glycine aminotransferase
ANT: adenine nucleotide transporter
ATP: adenosine triphosphate
Cr: creatine
CK: creatine kinase
CKB: brain creatine kinase
CKM: muscle creatine kinase
GAA: guanidinoacetic acid
GAMT: guanidinoacetate methyltransferase
MCT12: monocarboxylate transporter 12
mtCK: mitochondrial creatine kinase
smtCK: sarcomeric mitochondrial creatine kinase
PCr: phosphocreatine
ROS: reactive oxygen species
SAM: *S*-adenosylmethionine
SLC6A8: solute carrier family 6 member 8
